# Antinociceptive and Cytotoxic Activity of Opioid Peptides with Hydrazone and Hydrazide Moieties at the C-Terminus

**DOI:** 10.3390/molecules25153429

**Published:** 2020-07-28

**Authors:** Jolanta Dyniewicz, Piotr F. J. Lipiński, Piotr Kosson, Marta Bochyńska-Czyż, Joanna Matalińska, Aleksandra Misicka

**Affiliations:** 1 Department of Neuropeptides, Mossakowski Medical Research Centre Polish Academy of Sciences, Pawińskiego 5, 02-106 Warsaw, Poland; marta.bochynska@interia.eu (M.B.-C.); jmatalinska@imdik.pan.pl (J.M.); 2 Toxicology Research Laboratory, Mossakowski Medical Research Centre Polish Academy of Sciences, Pawińskiego 5, 02-106 Warsaw, Poland; pkosson@imdik.pan.pl; 3 Faculty of Chemistry, University of Warsaw, Pasteura 1, 02-093 Warsaw, Poland

**Keywords:** analgesics, cytotoxic activity, diacylhydrazine, µ-opioid receptor, multitarget compounds, *N′*-acylhydrazide, *N*-acylhydrazone, opioid peptides, peptidomimetics, privileged structures, structure activity relationships

## Abstract

In the present contribution, we analyze the influence that C-terminal extension of short opioid peptide sequences by organic fragments has on receptor affinity, in vivo analgesic activity, and antimelanoma properties. The considered fragments were based on either *N*-acylhydrazone (NAH) or *N′*-acylhydrazide motifs combined with the 3,5-bis(trifluoromethyl)phenyl moiety. Eleven novel compounds were synthesized and subject to biological evaluation. The analyzed compounds exhibit a diversified range of affinities for the µ opioid receptor (MOR), rather low δ opioid receptor (DOR) affinities, and no appreciable neurokinin-1 receptor binding. In three out of four pairs, *N*-acylhydrazone-based derivatives bind MOR better than their *N’*-acylhydrazide counterparts. The best of the novel derivatives have similar low nanomolar MOR binding affinity as the reference opioids, such as morphine and biphalin. The obtained order of MOR affinities was compared to the results of molecular docking. In vivo, four tested compounds turned out to be relatively strong analgesics. Finally, the NAH-based analogues reduce the number of melanoma cells in cell culture, while their *N*′-acylhydrazide counterparts do not. The antimelanoma properties are roughly correlated to the lipophilicity of the compounds.

## 1. Introduction

**Figure 1:**
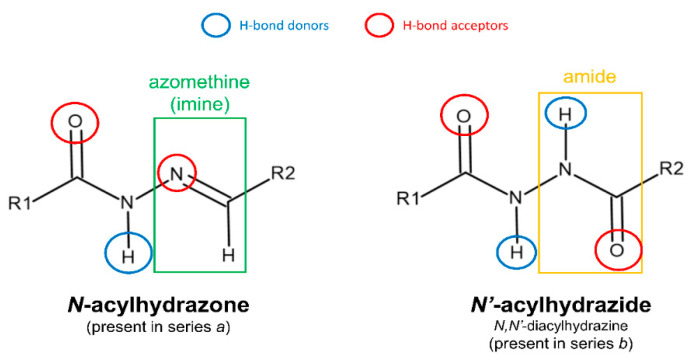
Comparison of *N*-acylhydrazone and *N*′-acylhydrazide structural motifs.

*N*-Acylhydrazone (NAH; otherwise termed hydrazide/hydrazone; Figure 1) has been identified as a “privileged structure” [1] that is a structural framework, which by being decorated with appropriate substituents is able to provide potent ligands for a variety of medicinally relevant molecular targets [2]. A few marketed drugs contain the *N*-acylhydrazone motif in their structures, e.g., azumolene, carbazochrome, dantrolene, nitrofurantoin, nitrofurazone, nifuroxazide, testosterone 17-enanthate 3-benzilic acid hydrazone [3]. Further compounds of this type, for example PAC-1 (a procaspase activator with antitumour action), are being currently considered in clinical trials (NCT02355535). Numerous other NAHs have been described and tested at the preclinical level, with some of them having potential to reach the clinic. The reported activities of *N*-acylhydrazone-bearing compounds include anti-inflammatory, analgesic, antithrombotic [4], antimicrobial [5,6], and antiproliferative (apoptosis-inducing) [7] properties. Other authors disclosed NAHs that were α-glucosidase inhibitors [8], non-ligand binding pocket androgen receptor antagonists [9], or histone deacetylases and phosphatidylinositol 3-kinases inhibitors [10]. A recent review on the roles of NAHs in modern medicinal chemistry was provided by Thota et al. [3].

A scaffold that is closely related to *N*-acylhydrazone is *N*′-acylhydrazide (*N*,*N*′-diacylhydrazine). Structurally, it differs from the former in having a second amide subunit instead of azomethine (Figure 1). R_1_ and R_2_ substituents attached at the ends of both these scaffolds are separated by an equal number of atoms; their positions may, however, be different for conformational reasons. While *N*-acylhydrazones are planar [9], the *N*′-acylhydrazides are usually twisted around the *N*-*N* bond [11], but planar structures have been also observed. Both scaffolds have distinct H-bonding opportunities. In *N*-acylhydrazones, there are two H-bond acceptor points (carbonyl oxygen and imine nitrogen) spaced by an H-bond donor (amide hydrogen). In *N*′-acylhydrazides, there are two acceptors (at the scaffold edges) and two donors (“inside” the motif). Due to the presence of an additional polar carbonyl group, *N*′-acylhydrazides are significantly less lipophilic than the corresponding *N*-acylhydrazones (by about 1.0 LogP unit). For the reasons mentioned above, consideration of the *N*-acylhydrazone/*N*′-acylhydrazide pairs of active compounds’ analogues is an interesting and rational approach for exploring structure-activity relationships (SAR).

Furthermore, both discussed fragments seem particularly well-suited for creating multitarget (multivalent), modular compounds of peptide or mixed peptide–organic structures. This is foremost due to facile synthesis, because hydrazones are readily obtained by “clicking” aldehydes with hydrazides (e.g., peptide hydrazides). *N*′-acylhydrazides could be produced by an equally simple reaction of a carboxylic acid (e.g., peptide) with a hydrazide (generated from another carboxylic acid). Examples of using hydrazone linkage to create modular conjugates include the work by Lipkowski et al., who joined oxymorphone and naltrexone with a portion of enkephalin or dynorphin A [12]. Ganguly et al. demonstrated the utility of the *N*-acylhydrazones for attaching the chelating moieties in radiopharmaceuticals [13]. The *N*′-acylhydrazide motif (usually talked about in terms of being a “hydrazide linker”) is present in a potent opioid analgesic with a dimeric structure, biphalin (H_2_N-Tyr-D-Ala-Gly-Phe-NH-NH←Phe←Gly←D-Ala←Tyr-NH_2_) [14,15]. This fragment is also found in numerous derivatives of biphalin [16,17,18,19,20], including the cyclic ones [21,22] that were obtained in the course of structure–activity investigations. Other dimeric opioids containing the *N*′-acylhydrazide substructure include dermorphin-based analogues [23]. 

Moreover, the hydrazide linker was applied to create multifunctional compounds based on the biphalin structure. For example, it served to append the opioid sequence with fluorescent elements, such as dansyl [24] or 7-succinylamido-4-methyl-coumarin [25] moieties. The hydrazide linker was also used in multitarget compounds having opioid receptor agonist and cholecystokinin receptor antagonist [26] pharmacophores. Yet another bivalent combination in which the hydrazide bridge found application was in hybrids of opioid agonist and neurokinin-1 receptor (NK1R) antagonists [27,28]. The rationale for preparing such multitarget compounds consisting of opioid sequences along with the pharmacophoric elements required for targeting some other receptors stems from the fact that simultaneous modulation of a few receptors involved in pain perception can provide analgesic compounds with enhanced efficacy and fewer side effects [29,30,31,32]. 

**Figure 2:**
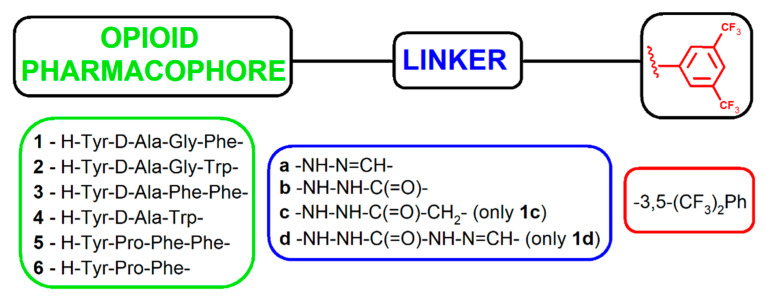
Structural variation of the reported compounds.

In the course of our works aimed at obtaining opioid receptor agonist/neurokinin-1 receptor antagonist chimeras, we designed a series of compounds (Figure 2, Table 1) in which opioid sequences (**1–6**) were appended (at the C-terminus) with the 3,5-bis(trifluoromethyl)phenyl moiety characteristic for neurokinin-1 antagonists. The structural elements that enabled this fusion were the discussed fragments: *N*-acylhydrazone (series **a**) and *N*′-acylhydrazide (series **b**). Moreover, in the case of H-Tyr-D-Ala-Gly-Phe- sequence (**1**), two additional linkers were considered (**c**, -NH-NH-C(=O)-CH_2_; and **d**, -NH-NH-C(=O)-NH-N=CH-). One of the compounds resulting from these efforts, JZ031 (**1a**), has been already described [33]. In the present contribution, we extend that report by discussing the synthesis, receptor binding, and molecular modelling of further 11 compounds. Moreover, the in vivo antinociceptive activity of compounds **1a**–**1d** was tested. Finally, the cytotoxic activity of the compounds under study was examined in a melanoma cell line, MeW155. 

## 2. Results and Discussion

### 2.1. Chemistry

**Scheme 1:**
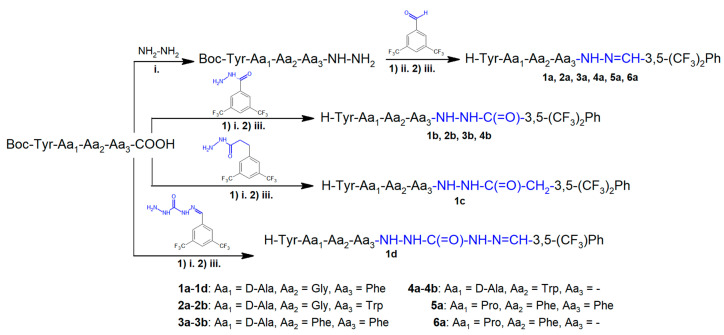
Synthesis of the reported compounds. Reagents and conditions: (i) *N*-hydroxysuccinimide, (HOSu, 1.1 equiv), *N*,*N*′-dicyclohexylocarbodiimide (DCC, 1 equiv), 1,1,3,3-tetramethylguanidine (TMG, 1.1 equiv), 24 h in dimethylformamide (DMF); (ii) 3,5-bis(trifluoromethyl)benzaldehyde, 20 h in isopropanol (i-PrOH); (iii) trifluoroacetic acid/ dichloromethane (TFA/DCM), 2 h.

The synthesis of the herein reported compounds (**1b** and **2a–6a**) was carried out by (1) obtaining the *N*-tert-butoxycarbonyl (Boc)-protected linear peptides in solution, (2) coupling them with the appropriate organic fragments (Scheme 1), and (3) removing the *N*-protection. 

In order to execute the second stage, for the analogues with the *N*-acylhydrazone motif in their structures (**2a**, **3a**, **4a**, **5a**, **6a**), the protected peptides were converted into hydrazides by reaction with hydrazine, and later the *N*-Boc-protected peptide hydrazides were condensed with 3,5-bis(trifluoromethyl)benzaldehyde. 

Alternatively, hydrazides of 3,5-bis-(trifluoromethyl) benzoic acid (for the synthesis of **1b**, **2b**, **3b**, **4b**) or 3,5-bis(trifluoromethyl)phenylacetic acid (for the synthesis of **1c**) were obtained and then reacted with *N*-Boc-protected peptides. Finally, in the case of **1d** analogue, the second stage was performed by the reaction of the *N*-Boc-protected peptide with the fragment obtained from condensation of 3,5-bis(trifluoromethyl)benzaldehyde with carbohydrazide. 

The purity and identity of the structures were confirmed by HPLC and ESI-MS, as well as by NMR. The analytical data for the synthesized analogues are summarized in Table 1. 

**Figure 3:**
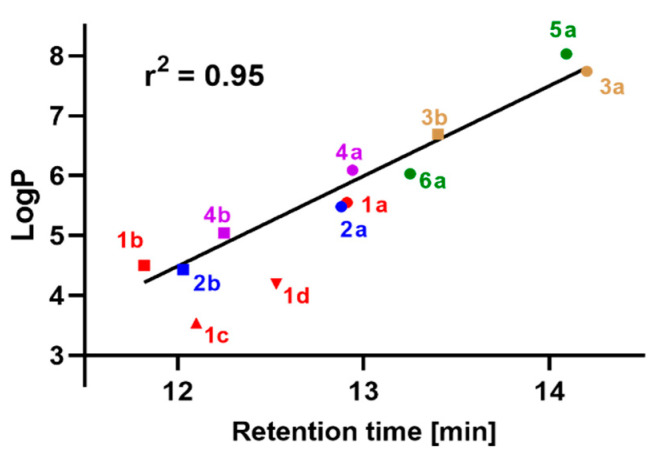
Correlation of RP-HPLC retention times of the studied compounds with theoretical LogP values. Compounds **1c** and **1d** excluded from the correlation. Points are colored so that analogues with the same peptide sequence have the same color.

RP-HPLC retention times of compounds **1a**–**6a** were plotted (Figure 3) against the theoretical lipophilicity descriptor, LogP, calculated in the ACD/ChemSketch program [34]. Upon excluding compounds **1c** and **1d**, a very good correlation appears, with a coefficient of determination of r^2^ = 0.95. This agreement of theoretical and experimental results confirms the additive nature of lipophilicity variation with exchanging *N*-acylhydrazone/*N*′-acylhydrazide motifs in our structures. Overall, the hydrazones are more lipophilic, with LogP values being greater by cca 1.0 unit and elution times greater by 0.7–1.1 min in the RP-HPLC system applied here. The remaining two linkers (present in **1c** and **1d**) represent intermediate levels of lipophilicity (in terms of the retention time; note that the LogP values do not fit this conclusion and counterintuitively suggest that **1c** and **1d** are even less lipophilic than **1b**.

### 2.2. Binding Affinity

The synthesized compounds were assayed for binding affinity to the μ- and δ-opioid (MOR and DOR, respectively), as well as to the neurokinin-1 receptors (NK1R). The determinations were performed in rat brain homogenates by competitive displacement of selective radioligands. The results are shown in Table 2 as the half-maximal inhibitory concentration (IC_50_) with the standard error of the mean (SEM). 

Contrary to the design assumptions, the majority of the compounds exhibit only very low NK1R binding (IC_50_ > 1000 nM). The sole exception is compound **3a**, which has a low NK1R affinity, with an IC_50_ value of 722.8 nM.

Regarding the opioid receptors, the presented compounds show a diversified spectrum of affinities. For MOR, the IC_50_ values are as different as a few nanomoles (**1c**, **1d**) and more than 1000 nM (**4b**). In three of the four *N*-acylhydrazone–*N*′-acylhydrazide analogue pairs (**1a** vs. **1b**, **3a** vs. **3b** and **4a** vs. **4b**), the hydrazones exhibit better MOR binding (4 to 10 times). In the fourth pair (**2a** vs. **2b**), the MOR affinities are almost equal for both analogues. 

If we compare the reported hydrazone compounds with the parent opioid sequences, for the enkephalin derivative (**1a**) expanding the C-terminus with 3,5-bis(trifluoromethyl)phenylhydrazone is rather neutral (or slightly negative) for affinity. This compound exhibits similar MOR affinity levels as its tetrapeptide amide counterpart (H-Tyr-D-Ala-Gly-Phe-NH_2_, K_i_ = 2.8 nM [35] or H-Tyr-D-Ala-Gly-Phe-NH-NH_2_, IC_50_ = 4.7 nM [36]). For the enkephalin-based derivative with Trp in the 4th position (**2a**), the IC_50_ reading 112.01 nM can be considered a slight decrease in affinity compared to the parent tetrapeptide, as H-Tyr-D-Ala-Gly-Trp-NH_2_ was reported to be an about 2 times weaker as a MOR binder than the [Phe^4^]-variant [37], while for the hydrazones **2a** and **1a** the IC_50_ ratio is about 10.

TAPP-based hydrazone (**3a**; TAPP = H-Tyr-D-Ala-Phe-Phe-NH_2_) binds with IC_50_ = 90.04 nM, which is clearly a higher value than that recently determined for the tetrapeptide in our laboratory (IC_50_ = 5.1 nM [38]). Regarding compound **4a**, a similar comparison is not straightforward, as we are not aware of any MOR binding data for amide H-Tyr-D-Ala-Trp-NH_2_. On the other hand, Laskowska et al. [39] reported that tripeptide H-Tyr-D-Ala-Trp-OH has low MOR affinity with IC_50_ = 977 nM, while expanding this structure by attaching *trans*-1-cinnamylpiperazine (Cyn) improves the binding, so that H-Tyr-D-Ala-Trp-Cyn has an IC_50_ value of 77.6 nM. In this case **4a**, whose IC_50_ value is 118.11 nM, could be considered to enjoy the very same improvement of affinity upon attaching the 3,5-bis(trifluoromethyl)phenylhydrazone element to H-Tyr-D-Ala-Trp- sequence. The endomorphin-2-based analogue (**5a**) has a slightly worse MOR affinity (IC_50_ = 28.5 nM) than that of the parent tetrapeptide (IC_50_ = 3.9 nM [40]). The C-truncated EM-2 (H-Tyr-Pro-Phe-NH_2_) was previously found to have a MOR binding affinity with an inhibition constant K_i_ of 46.3 [41]. Thus, in the case of this sequence, attachment of 3,5-bis(trifluoromethyl)phenylhydrazone (**6a**, IC_50_ = 113.1 nM) seems to be slightly adverse to MOR affinity.

The highest MOR affinities in our set were found for compounds **1c** and **1d**, in which the C-terminal part is expanded by organic fragments containing *N*′-acetylhydrazide (**1c**) or -C(=O)-NH-NH-C(=O)-NH-N=CH- substructures (**1d**). Particularly interesting is that **1c**, being only one methylene unit longer than **1b**, exhibits significantly better MOR binding (approximately 21 times lower IC_50_ value).

For DOR, half of the compounds exhibit low affinity, with IC_50_ > 1000 nM. As could be expected of endomorphin-2- and TAPP-based derivatives, compounds **3a**, **3b**, **5a**, and **6a** do not exhibit any appreciable DOR binding. In the enkephalin-based analogues, the type of C-terminal expansion modifies the DOR affinity, which for the H-Tyr-D-Ala-Gly-Phe-NH_2_ was reported to be rather moderate (K_i_ = 300 nM [35]). The hydrazone **1a** shows IC_50_ = 74.2 nM (previously reported [33]). The analogue with *N*′-acylhydrazide (**1b**) has a significantly lower DOR binding value (IC_50_ = 413.45 nM). Interestingly, compounds **1c** and **1d**, which exhibit similarly high MOR affinity, differ with respect to DOR binding. For the former, the determined DOR IC_50_ value is 161.65 nM, while for the latter the value is 44.04 nM. Enkephalin-related compounds with Trp in the 3rd position exhibit low or at best moderate DOR affinity (DOR IC_50_ values of 914.4 nM and 467.05 nM for hydrazone **2a** and N′-acylhydrazide **2b**, respectively).

In general, the most potent MOR ligands reported here (**1c** and **1d**) have similarly high affinity as typical reference opioids, be they peptides (like biphalin, MOR IC_50_ = 1.4 nM [33]) or non-peptides (like morphine, IC_50_ = 4.02 nM [42]). 

### 2.3. Molecular Docking

In order to rationalize the observed binding data, the reported compounds were modelled in the µ-opioid receptor binding site. The modelling was done by building the *N*-acylhydrazone-, *N*′-acylhydrazide-, or *N*′-acetylhydrazide-based fragments into the peptide structures in the MOR binding site (PDB accession code: 6DDF [43]), followed by local search docking using AutoDock 4.2.6 [44]. In the body of the paper we describe a few representative compounds (**1a**, **1b**, **1c**, **3a**, **3b**, **4a**, **4b**), while the results for the remaining analogues are given in the Supplementary Materials. The Supplementary Materials also contain the description of the redock validation exercise with AutoDock 4.2.6 and AutoDock Vina [45], as well as the comparison of modelling compounds **1a**–**6a** executed with AutoDock Vina.

**Figure 4:**
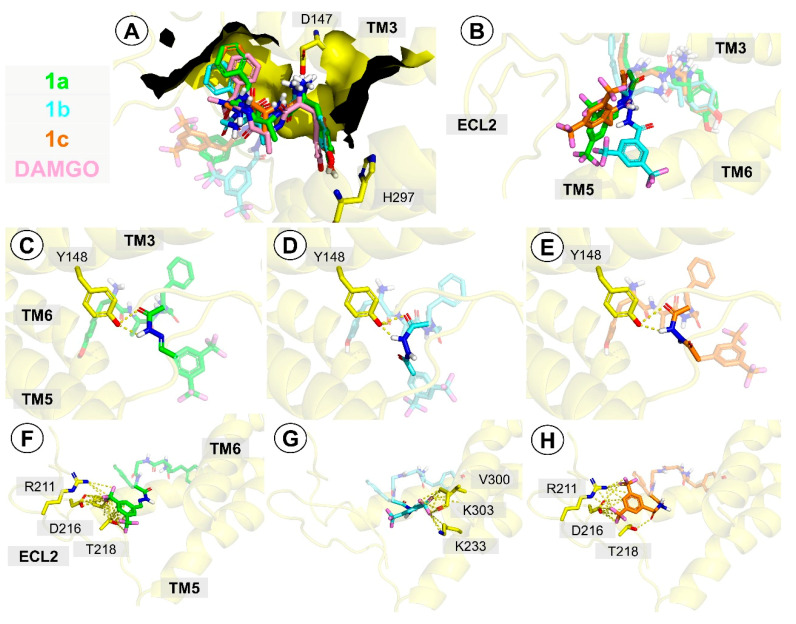
Binding modes of compounds **1a** (green), **1b** (aquamarine blue), and **1c** (orange) in the MOR binding site (yellow). The ligands are shown as colored sticks. Several residues and transmembrane helices (relevant for each picture) are shown and labelled. Yellow dotted lines represent interactions. (**A**) All of the compounds superimposed on DAMGO (pink, from 6DDF [43]). (**B**) Focus on the C-terminal part and compounds **1a**–**1c** together. (**C**–**E**) Focus on the interactions with Y148 and compounds **1a** (**C**), **1b** (**D**), and **1c** (**E**). (**F**–**H**) Focus on the C-terminal part and compounds **1a** (**F**), **1b** (**G**), and **1c** (**H**).

In the case of compounds **1a**–**1c**, the starting position of the peptide sequence (H-Tyr-D-Ala-Gly-Phe-) was based on the position of H-Tyr-D-Ala-Gly-*N*-MePhe-Gly-ol (DAMGO) in the experimental structure (PDB accession code: 6DDF [43]). As a result of the local docking search for our derivatives, only minor displacements occurred in the peptide part (Figure 4A), and overall the positioning of this part was close to that of the corresponding fragments in DAMGO (6DDF [43]). The main features of this binding mode are (1) the canonical ionic interaction of the protonated Tyr^1^ amine with D147, (2) the location of the Tyr^1^ phenol group close to H297, and (3) the position of the Phe^4^ in a hydrophobic subsite made of side chains of several residues belonging to transmembrane helix 3 (TM3) and extracellular loops 1 and 2 (ECL1 and ECL2). The first and the third features correspond to analogous elements of the binding modes of small molecular MOR agonist BU72 (as found in crystal structure 5C1M [46]) or fentanyl (as found by modelling [42,47]).

Regarding the C-terminal part (Figure 4B), all three compounds (**1a**–**1c**) assume the *cis*-geometry of the amide joining the peptide and the organic fragment (Figure 4C–E). This allows for the creation of two hydrogen bonds with the Y148 phenol group. In the case of **1a**, the flat hydrazone fragment (with a co-planar ring and double bond) reaches towards the residues of ECL2 (Figure 4F). The aromatic ring is placed close to T218, while the fluorines of the CF_3_ groups interact with D216 carboxylate and C217 carbonyl groups or with T218 hydroxyl and R211 guanidine moieties. On the other hand, in **1b** the twisted N(H)-NH bond causes the 3,5-bis-CF_3_-Ph to locate closer to TM6 (Figure 4G). One of the CF_3_ groups interacts with K233, K303, and V300, while the other one is exposed to the solvent. The ring does not seem to be involved in the interactions with the binding site residues either. In the case of **1c**, elongation of the fragment by one methylene (with free rotation) again allows for contact with the ECL2 (Figure 4H). The hydrogen in the hydrazides’ *N*′-nitrogen participates in hydrogen bonding to T218. Both CF_3_ groups form interactions with R211 and D216. 

Overall, these differences in the binding modes of the C-terminal parts of **1a**–**1c** form a reasonable basis for explaining (qualitatively) the experimental differences in affinity. Let us note that the binding model of **1a** considered here is different than that we reported previously [33]. The differences are with respect to both the position of Phe^4^, as well as the position of the 3,5-bis(trifluoromethyl)phenylhydrazone element. We decided to embrace this novel model due to its consistency with the experimental position of DAMGO.

**Figure 5:**
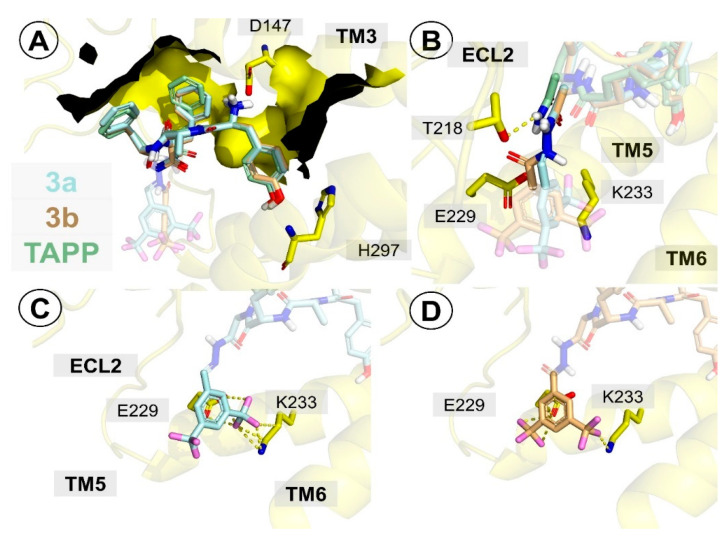
Binding modes of compounds **3a** (palecyan) and **3b** (light orange) in the MOR binding site (yellow). The ligands are shown as colored sticks. Several residues and transmembrane helices (relevant for each picture) are shown and labelled. Yellow dotted lines represent interactions. (**A**) Both compounds superimposed on TAPP (pink, from 6DDF [43]). (**B**) Focus on the interactions with T218 and compounds **3a**, **3b**, and TAPP together. (**C**,**D**) Focus on the interactions with E229 and K233, as well as compounds **3a** (**C**), **3b** (**D**), and **1c** (**E**).

Regarding the TAPP-based analogues (**3a** and **3b**), the starting position of the peptide sequence (H-Tyr-D-Ala-Phe-Phe-) was based on the position of TAPP reported in our recent paper, which focused on TAPP derivatives [38]. Again, as a result of the local docking search performed for the novel analogues, only minor displacements occurred in the peptide part (Figure 5A). The main features of the binding mode of TAPP (**3a** and **3b**) for the peptide fragment are: (1) the canonical ionic interaction of protonated Tyr^1^ amine with D147; (2) the location of the Tyr^1^ phenol group close to H297; (3) the position of the Phe^4^ in a hydrophobic subsite made of side chains of several residues belonging to TM3, ECL1, and ECL2; and (4) the position of Phe^3^ close to N127 and H319. The first three features are identical to those found in the enkephalin-based series described above. Moreover, TAPP has been predicted to form a hydrogen bond to T218 via the C-terminal amide (Figure 5B). For **3a** and **3b**, in which this amide joins the peptide and the organic fragment, the need to accommodate the organic fragment seems to impede formation of this hydrogen bond (or at least to create a certain distortion in the interaction geometry). This corresponds well to the experimentally determined decrease in affinity for **3a** and **3b** in comparison to the parent TAPP. The aromatic rings in both analogues are located close to TM5. The CF_3_ groups form interactions with E229 and K233 (Figure 5C,D).

**Figure 6:**
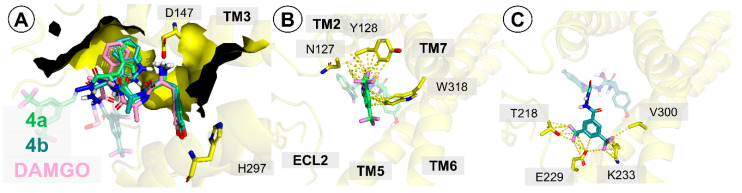
Binding modes of compounds **4a** (lime) and **4b** (light teal) in the MOR binding site (yellow). The ligands are shown as colored sticks. Several residues and transmembrane helices (relevant for each picture) are shown and labelled. Yellow dotted lines represent interactions. (**A**) Both compounds superimposed on DAMGO (pink, from 6DDF [43]). (**B**,**C**) Focus on the on the interactions of the C-terminal part, showing compounds **4a** (**B**) and **4b** (**C**).

For modelling of compounds **4a** and **4b**, the tripeptide amide (H-Tyr-D-Ala-Trp-NH_2_) was first docked and then the organic fragments were appended to the structure from docking, as with the former compounds. After the local optimization, the obtained binding mode was common to **4a** and **4b** in the peptide part, as expected. Again, the canonical ionic interaction of protonated Tyr^1^ amine with D147 was formed and the Tyr^1^ phenol group was positioned close to H297, just as in the case of enkephalin-based and TAPP-based derivatives (Figure 6A). The indole ring of Trp^3^ assumed a position close to TM3 residues I144, V143, and Y148. The phenol group of the latter is predicted to form a hydrogen bond to the indole’s nitrogen. According to modelling, the 3,5-bis(trifluoromethyl)phenyl fragments of both compounds are located in different subareas of the binding site. In the hydrazone-based derivative (**4a**), the moiety approaches TM2 and TM7 (Figure 6B). This allows the CF_3_ groups to contact Y128 and N127. Moreover, the aromatic ring is involved in hydrophobic interactions with W318. In the hydrazide-based analogue (**4b**), the organic part is directed towards ECL2, TM5, and TM6 (Figure 6C). The CF_3_ groups interact with the sidechains of T218, K233, E229, and V300. Finally, considering all the modelled compounds (**1a**, **1b**, **1c**, **3a**, **3b**, **4a**, **4b**, DAMGO, and TAPP), it should be noted that an attempt to quantitatively correlate the affinities with the predicted energies in the whole set gives a very poor correlation (Figure SM-1).

### 2.4. In Vivo Analgesic Activity

**Figure 7:**
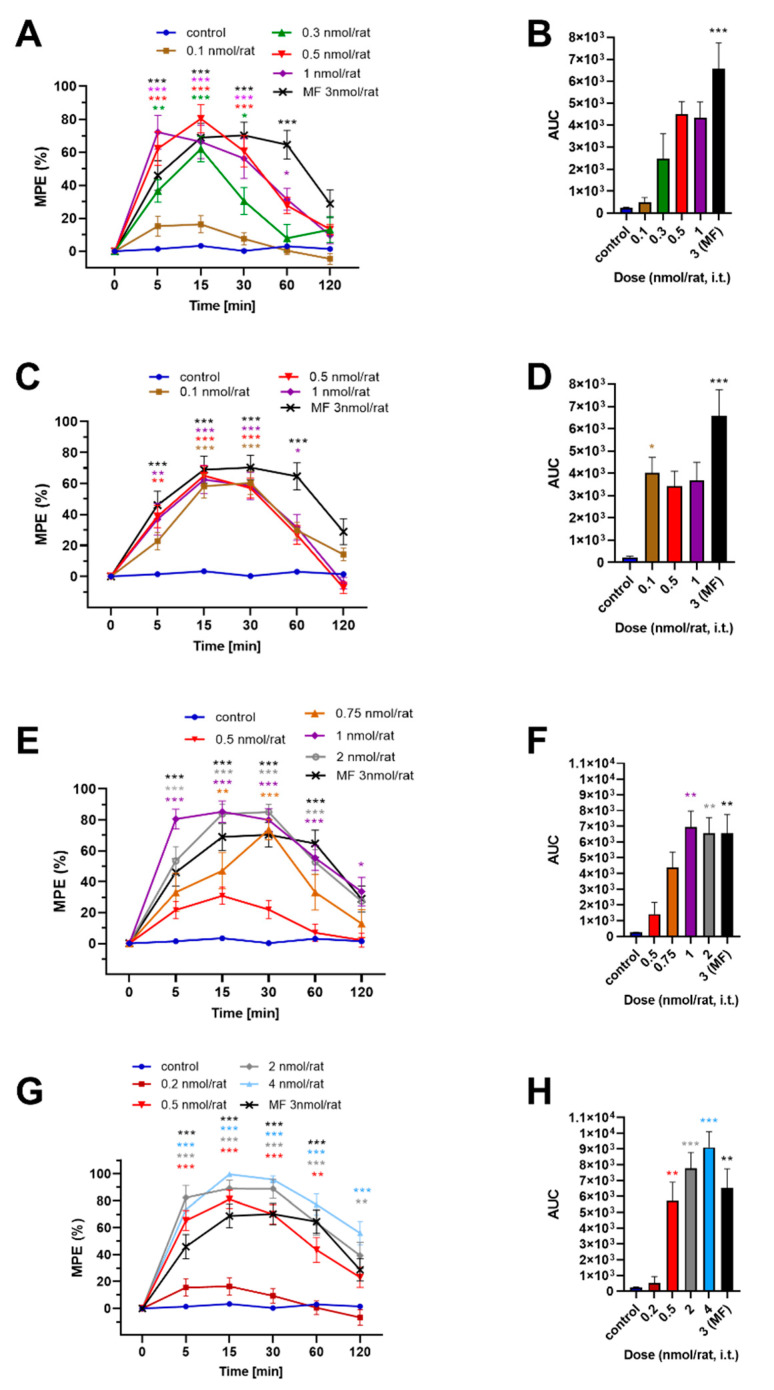
Analgesic responses of compounds **1a** (**A**,**B**), **1b** (**C**,**D**), **1c** (**E**,**F**), and **1d** (**G**,**H**), as well as morphine (MF). In the left column given is the time course curve of the effect. In the right column are column plots of the area under the time course curve (AUC) for different doses. Results were either analyzed with two-way ANOVA followed by the Bonferroni post-hoc test (**A**,**C**,**E**,**G**) or one-way ANOVA followed by the Dunnett post-hoc test (**B**,**D**,**F**,**H**) and plotted as the mean ± SEM (*n* = 4–8). Asterisks (*) indicate a significant difference compared with saline-injected animals. Note: * *p* < 0.033, ** *p* < 0.002, and *** *p* < 0.001.

**Figure 8:**
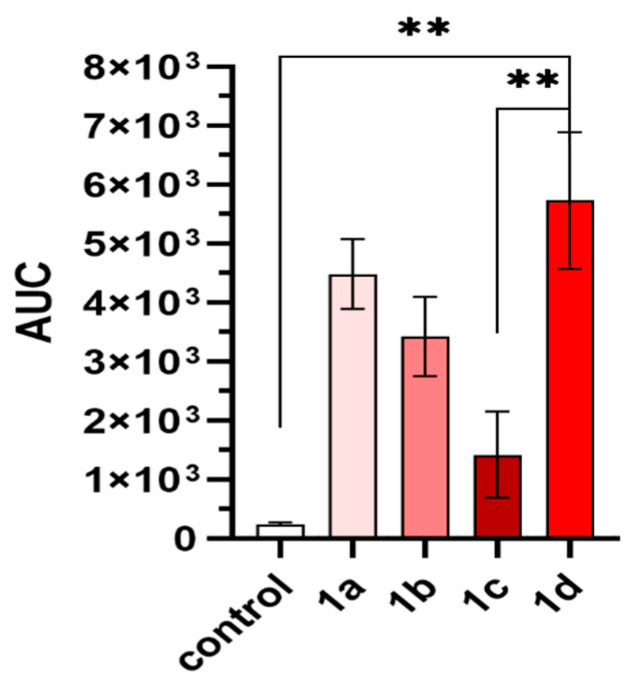
Comparison of the area under the time course curve (AUC) with a 0.5 nmol/rat dose for compounds **1a**–**1d**. Results were analyzed with one-way ANOVA followed by the Tukey post-hoc test. Asterisks (*) indicate a significant difference on comparison of particular pair of columns. Note: * *p* < 0.033, and ** *p* < 0.002.

Four of the reported compounds (**1a**–**1d**) based on the H-Tyr-D-Ala-Gly-Phe- sequence were tested in vivo for antinociceptive activity in male Wistar rats in a tail flick test after the intrathecal (i.t.) administration. The data for compound **1a** have already been published [33]. The results are presented in Figure 7 and Figure 8 and Table 3. 

All studied compounds exhibited time-dependent analgesic effects. The effects were also dose-dependent, except for compound **1b**, for which doses of 0.1 nmol/rat, 0.5 nmol/rat, and 1.0 nmol/rat produced similar results. Comparing the analgesic action of the compounds administered in 0.5 nmol/rat dose, all four analogues reach their peak activity 15 min after the injection. At this time point and with this dose, the highest effect was observed for **1a** and **1d** (~ 80% of maximal possible effect, MPE). The lowest value was found for compound **1c** (31 ± 6% MPE), while the analogue **1b** exhibited 65 ± 6% MPE. At 120 min post-injection (0.5 nmol/rat), none of the compounds had an analgesic effect significantly different than the control, although the plot for **1d** seemed to “tail” towards the right side, suggesting this compound may have longer duration of action. In fact, for a 2 nmol/rat dose, as much as 39 ± 10% MPE can be measured for **1d**, even at 120 min after administration. If the area under the time course curve (AUC, Figure 7, the column on the right) is considered, compounds **1a**, **1b**, and **1d** had similar AUC values (for 0.5 nmol/rat dose) of 4483 ± 592, 3421 ± 673, and 5732 ± 1161 units, respectively. The same parameter for **1c** was significantly lower (1417 ± 730.1). 

The highest antinociceptive effects found for each compound were 80 ± 9% MPE (15 min, 0.5 nmol/rat, **1a**), 65 ± 6% MPE (15 min, 0.5 nmol/rat, **1b**), 85 ± 7% MPE (15 min, 1 nmol/rat, **1c**), 99 ± 7% MPE (15 min, 4 nmol/rat, **1d**). In terms of the effective dose (ED_50_, Table 3), the hydrazone **1a** was found to be the most active (ED_50_ = 0.18 ± 0.05 nmol/rat). For the analogues **1c** and **1d**, the values were 0.56 ± 0.04 and 0.32 ± 0.04, respectively. 

Overall, the antinociceptive action (in the tail flick test, after i.t. administration) of the tested compounds was relatively high, comparable to that we have previously found for morphine (3 nmol/rat) in the same test [33] (in terms of MPE and the peak activity time point)**.** On the other hand, the observed antinociception was weaker than in the case of the potent, dimeric opioid peptide biphalin [48], for which lower doses than tested here were enough to exert similar activity. It should be noted that while these good antinociceptive effects of **1a**, **1c**, and **1d** are what one would expect of high-affinity opioid agonists, the analgesic efficacy of the compound **1b** is somehow anomalous, given that this analogue has the weakest MOR and DOR affinities out of the **1a**–**1d** subseries. Perhaps an action via some receptor other than those tested herein is responsible for this effect. This speculation requires further investigation. Finally, let us note that the lack of the dose dependency for compound **1b** could stem from the fact that perhaps all the tested doses were at the plateau of the dose–response curve. In order to verify this, the in vivo determination ought to be done with a wider range of concentrations.

### 2.5. Cytotoxicity

The neurokinin-1 receptor antagonists are known to exhibit cytotoxic effects [49,50,51,52], in particular in cancers, as many types of cancer cells overexpress the NK-1R. These effects were found both in vitro and in vivo. 

**Figure 9:**
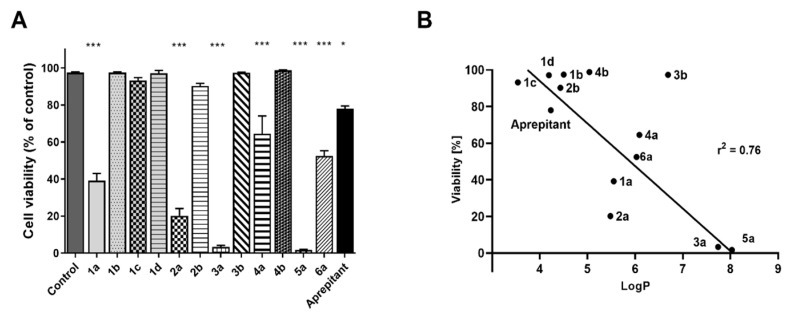
The results of cytotoxicity evaluation. (**A**) Viability of MeW155 cells after incubation with the tested compounds (as % of the control value). The results were analyzed with repeated measures one-way ANOVA followed by the Dunnett post-hoc test. Asterisks (*) indicate a significant difference compared to control. Note: * *p* < 0.033, and *** *p* < 0.001. (**B**) Correlation of melanoma cell line MeW155 viability (%) with the theoretical LogP value. Point **3b** excluded from correlation.

Contrary to the design assumptions, our compounds do not have high NK1R affinity. Still, since we have recently found [53] that a chimeric opioid-antitachykinin peptide, AWL3020, exhibits some significant cytotoxicity in a few cancer cell lines, despite having a very low NK1R affinity (IC_50_~70 µM), we tested the influence of compounds **1a**–**6a** on human melanoma cells. The determination was performed by counting the cells of the MeW155 cell line after incubating them with 50 µM of the tested compounds. The results (presented as % of the control value) are shown in Figure 9A.

It turned out that all *N*-acylhydrazone-based derivatives (**1a**, **2a**, **3a**, **4a**, **5a**, **6a**) significantly decrease the number of melanoma cells. On the other hand, the analogues with *N*′-acylhydrazide motif (**1b**, **2b**, **3b**, **4b**) display no effect. Similarly, no effect was found for the longest derivatives (**1c** and **1d**). The largest decrease in cell numbers was found for analogues **3a** and **5a**, for which only a few intact MeW155 cells were observed under the microscope (~ 2 % of the control value). For compounds **2a**, **1a**, **4a** and **6a**, the following values were determined: 20 ± 4%; 39 ± 4%; 65 ± 10%; 53 ± 3% of the control, respectively. 

Interestingly, the cytotoxic action of compounds **3a** and **5a** (and furthermore also of **2a**, **1a**, and **6a**) is significantly stronger than that of aprepitant (78 ± 4%), reinforcing the notion that the observed activity is not associated with the NK1R. Moreover, these compounds influence the melanoma cells significantly more strongly than the mentioned AWL3020 compound, for which the very same type of assay found 88% of the control value at 50 µM (this analogue was much more potent in other cell lines).

The ordering of the cytotoxic effects found for our analogues is not associated with either MOR or DOR affinities. Some imperfect relationship may be found between these cytotoxic effects and LogP (Figure 9B). For such a correlation, upon exclusion of **3b**, the coefficient of determination is equal to 0.76. Roughly, the more lipophilic the compound, the more it decreases the number of cells. This may be indicative of some non-specific (non-receptor-mediated) cytotoxic effects being at work here. Otherwise, it is also possible that the observed cytotoxic effects of the hydrazones could be exerted via some intracellular (and not a membrane one) molecular target, e.g., a receptor. In that case, a factor limiting the activity (irrespective of the affinity for this hypothetical target) could be the ability of the compounds to cross the cellular membrane. A speculative candidate for such a target may be the opioid growth factor receptor (OGFr), which binds [Met^5^]-enkephalin (also called opioid growth factor, OGF), and upon the interaction with this opioid peptide inhibits proliferation of cells [54]. Further research shall be required to investigate this possibility. Furthermore, the cytotoxicity should be tested on other cancer cell lines, as well as on normal cell lines, in order to determine the selectivity of the compounds. Another important step will be assaying the effect on cells that the tetrapeptide amides and 3,5-bis-CF_3_-Ph fragments have on their own.

## 3. Materials and Methods

### 3.1. Chemistry

All amino acids derivatives, reagents, and solvents were obtained from commercial suppliers and were used without further purification. *N*-Boc-protected compounds were purified on Merck 230–400 mesh silica gel 60 (Merck, Poland) by column chromatography. TLC analyses were carried out on silica gel plates (silica gel 60 F254, Merck, Poland); observed using UV light and visualized with 1% ninhydrin solution in MeOH. The target compounds (**1b**–**6a**) were purified by RP HPLC on a Merc Hitachi HPLC system with a reverse-phase column KROMASIL C8 (20 × 250 mm). The mobile phase was 0.1% trifluoroacetic acid (TFA) in water/acetonitrile. The obtained compounds were analyzed on a Shimadzu LC-MS system (Shim-pol, Poland) with a Jupiter 4 µm Proteo 90 Å (250 × 4.6 mm, 4 µm) column using a solvent system containing 0.05% formic acid (FA) in water/acetonitrile. For selected compounds, nuclear magnetic resonance (NMR) spectra (1H, 13C, DEPT, COSY, HSQC, HMBC) were also recorded with a Varian VNMRS at 600 MHz. 

### 3.2. Synthesis

*N*-Boc-protected peptide fragments (as carboxylic acids) were obtained according to solution phase strategy described previously by Lipkowski [55] and were used in subsequent steps without further purification. 

#### 3.2.1. General Method for Synthesis of Intermediate Hydrazides

*N*-Boc-protected peptide, 3,5-bis(trifluoromethyl)phenylacetic acid or 3,5-bis(trifluoromethyl)benzoic acid (1 equiv) were dissolved in dimethylformamide (DMF), *N*-hydroxysuccinimide (HOSu) (1.1 equiv) was added, then the mixture was cooled to 0–5 °C. Further, *N*,*N*′-dicyclohexylocarbodiimide (DCC) (1 equiv) was added and the reaction mixture was stirred for 30 min with cooling, then for 2 h at room temperature. Then, hydrazine hydrate (10 equiv) was added and the reaction mixture was stirred for 20 h. After filtering the *N*,*N*′-dicyclohexylourea (DCU) precipitate, the filtrate was concentrated under reduced pressure. The resulting products were precipitated by adding 15% KHCO_3_, then were filtered off, washed with 5% KHCO_3_ and water, dried, and used without further purification. The yields of crude products were 46–97%.

#### 3.2.2. General Method for Synthesis of *N*-acylhydrazone-Type Analogues (**2a, 3a, 4a, 5a, 6a**)

To the solution of 3,5-bis-(trifluoromethyl)benzaldehyde (1 equiv) in isopropanol (i-PrOH) an appropriate *N*-protected peptide hydrazide (1 equiv) was added, then the reaction was left overnight. The resulting precipitate was filtered off (or alternatively, the solvent was evaporated) under reduced pressure. The precipitate was washed with water and dried. The product was purified on silica gel with the appropriate solvent mixtures (ethyl acetate/hexane or ethyl acetate/methanol; exact compositions are given in Supplementary Materials). Yields after purification were 20–60%. 

The same procedure was used for the synthesis of monocarbohydrazone (yield = 54%) of 3,5-bis-(trifluoromethyl)benzaldehyde required for the synthesis of compound **1d**. Monocarbohydrazone was recrystallized from water/ethanol (1:1).

#### 3.2.3. General Method for Synthesis of Hydrazide Type of Analogues (**1b, 1c, 1d, 2b, 3b, 4b**)

An appropriate *N*-protected peptide (1 equiv) was dissolved in DMF, HOSu (1.1 equiv) was added, then the mixture was cooled to 0–5 °C. Then, DCC (1 equiv) was added and the reaction mixture was stirred for 30 min with cooling and for 2 h at room temperature. To the reaction mixture an appropriate hydrazide (1.1 equiv) solution in DMF with 1,1,3,3-tetramethylguanidine (TMG) (1.1 equiv) was added and the reaction was left for 20 h. Precipitated *N*,*N′*-Dicyclohexylurea (DCU) was filtered off, the filtrate was condensed under reduced pressure, and the residue was poured into 15% citric acid. The precipitate was filtered off and washed with 5% citric acid and water. If the product did not precipitate, it was isolated by extraction with ethyl acetate. The organic phase was washed with 5% citric acid (3×) and water (3×), dried with anhydrous MgSO_4_, then the solvent was evaporated. The product was purified on silica gel using proper solvents (ethyl acetate/hexane or ethyl acetate/methanol; exact compositions are given in Supplementary Materials). Yields after purification were between 16 and 42%.

The *N*-protected precursors obtained by the above procedures were next deprotected by TFA, and the resulting crude compounds were purified by preparative HPLC if necessary and analyzed by ESI-MS (see Table 1) and NMR (details in Supplementary Materials). Representative examples of NMR assignments for compounds containing each type of C-terminal organic fragment are given below. The remaining NMR data for five other compounds are given in the Supplementary Materials.

Compound **1b** (H-Tyr-D-Ala-Gly-Phe-NH-NH-C(=O)-3,5-(CF_3_)_2_Ph)

^1^H NMR(600 MHz, DMSO) δ: 10.48 (d, J = 2.32 Hz, 2H, NH-NH), 9.35 (br. s, 1H, OH-Tyr), 8.52 (1H, NH-Ala), 8.51 (1HAr), 8.38 (s, 1HAr), 8.29 (2H, NH, Tyr), 8.21 (d, J = 8.62 Hz, 1H, NH-Phe), 8.16 (t, J = 5.97Hz, 1H, NH-Gly), 7.24–7.29 (m, 3Hδ,ε-Phe), 7.28 (1HAr), 7.18 (2Hε-Phe), 6.99 (d, J = 8.63 Hz, 2Hδ-Tyr), 6.67 (d, J = 8.62 Hz, 2Hε-Tyr), 4.66 (J = 3.32 Hz, 1Hα-Phe), 4.30 (m, J = 7.30 Hz, 1Hα-Ala), 3.96 (1Hα-Tyr), 3.74 (d, J = 5.97 Hz, 1Hα-Gly), 3.59 (d, J = 5.64 Hz, 1Hα-Gly), 3.09 (1Hβ-Phe), 2.83 (1Hβ–Phe), 2.81 (2Hβ–Tyr), 1.02 (d, J = 6.8 Hz, 3Hβ-Ala).

^13^C NMR(150 MHz, DMSO) δ: 172.2 (C=O, Ala), 171.0 (C=O, Ar), 171.0 (C=O, Phe), 168.8 (C=O, Gly), 162.9 (C=O, Tyr), 157.1 (Cζ–Tyr), 138.0 (Cγ–Phe), 131.1 (Cδ–Tyr), 129.8 (Cζ–Phe), 129.8 (2Cζ–Phe), 128.8 (CAr), 126.9 (Cε–Phe), 126.2 (CAr), 125.2 (C–Tyr), 124.4 (CAr), 122.6 (CAr), 115.8 (Cε–Tyr), 54.2 (Cα–Tyr), 53.1 (Cα–Phe), 48.6 (Cα–Ala), 41.9 (Cα–Gly), 38.4 (Cβ–Phe), 36.8 (Cβ–Tyr), 19.4 (Cβ–Ala).

Compound **1c** (H-Tyr-D-Ala-Gly-Phe-NH-NH-C(=O)-CH_2_- 3,5-(CF_3_)_2_Ph)

^1^H NMR(600 MHz, DMSO) δ: 10.50 (2H, NH-NH), 9.33 (br. s, 1H, OH-Tyr), 8.55 (1HAr), 8.40 (1H, NH-Ala), 8.20 (2H, NH-Tyr), 8.10 (m, 1H, NH-Phe), 8.20 (m, 1H, NH-Gly), 8.03 (1HAr), 7.96 (1HAr), 7.20–7.27 (m, 5HAr-Phe), 7.00 (2Hδ-Tyr), 6.66 (2Hε-Tyr), 4.60 (1Hα-Tyr), 4.30 (q, J = 6.9 Hz, 1Hα-Ala), 4.01 (1Hα-Phe), 3.89 (s, 2H, C(=O)-CH_2_-), 3.50–3.80 (2Hα-Gly), 3.02 (d, J = 3.8 Hz, 1Hβ-Phe), 2.89 (1Hβ–Tyr), 2.86 (1Hβ–Tyr), 2.87 (d, J = 3.8 Hz, 1Hβ–Phe), 1.05 (d, J = 6.7 Hz, 3Hβ-Ala). 

^13^C NMR (150 MHz, DMSO) δ: 172.3 (C=O, Ala), 171.9 (C=O, Ar), 170.7 (C=O, Gly), 168.2 (C=O, Phe), 167.0 (C=O, Tyr), 156.7 (C ζ–Tyr), 137.8 (C γ–Phe), 130.6 (C δ–Tyr), 129.5 (2C δ–Phe), 128.0 (CAr), 127.2 (CAr), 126.8 (Cζ–Phe), 124.9 (Cγ–Tyr), 124.5 (CAr), 121.0 (CAr), 115.6 (Cε–Tyr), 53.6 (Cα–Tyr), 52.4 (Cα–Phe), 47.8 (Cα–Ala), 41.2 (Cα–Gly), 38.1 (Cβ–Phe), 35.7 (Cβ–Tyr), 19.1 (Cβ–Ala).

Compound **1d** (H-Tyr-D-Ala-Gly-Phe-NH-NH-C(=O)-NH-N=CH- 3,5-(CF_3_)_2_Ph)

^1^H NMR(600 MHz, DMSO) δ: 9.96 (2H, NH-NH), 9.33 (br. s, 1H, OH-Tyr), 8.55 (1HAr), 8.53 (1H, NH-N=), 8.52 (1H, N=CH), 8.51 (1H, NH-Ala), 8.22 (2H, NH-Tyr), 8.15 (m, 1H, NH-Phe), 8.14 (m, 1H, NH-Gly), 8.04 (1HAr), 7.27 (1HAr), 7.27 (d, J = 7.3 Hz, 2Hδ-Phe), 7.24 (t, J = 7.3 Hz, 2Hε-Phe), 7.17 (t, J = 7.02, 1Hζ-Phe), 6.99 (d, J = 8.54 Hz, 2Hδ-Tyr), 6.67 (d, J = 8.55 Hz, 2Hε-Tyr), 4.62 (1Hα-Phe), 4.30 (q, J = 7.02 Hz, 1Hα-Ala), 3.96 (t, J = 7.6 Hz, 1Hα-Tyr), 3.56–3.72 (2Hα-Gly), 3.07 (1Hβ-Phe), 2.92 (1Hβ–Tyr), 2.88 (1Hβ–Tyr), 2.83 (1Hβ–Phe), 1.03 (d, J = 7.02 Hz, 3Hβ-Ala). 

^13^C NMR (150 MHz, DMSO) δ: 171.7 (C=O, Ala), 171.7 (C=O, Phe), 171.2 (C=O, Ar), 168.1 (C=O, Gly), 167.6 (C=O, Tyr), 156.5 (Cζ–Tyr), 137.7 (C–N=C), 137.7 (Cγ–Phe), 130.7 (Cδ–Tyr), 129.1 (2Cδ–Phe), 128.4 (2Cε–Phe), 128.0 (CAr), 127.2 (CAr), 126.3 (Cζ–Phe), 125.1 (Cγ–Tyr), 127.2 (CAr), 122.3 (CAr), 115.1 (Cε–Tyr), 53.4 (Cα–Tyr), 52.3 (Cα–Phe), 48.5 (Cα–Ala), 41.8 (Cα–Gly), 38.4 (Cβ–Phe), 36.7 (Cβ–Tyr), 18.3 (Cβ–Ala).

### 3.3. Binding Affinity Determinations

The binding affinity of compounds **1b**–**6a** for MOR, DOR, and NK1R was determined in competitive radioligand binding assays according to the method previously described [23,53]. The specific radioligands were [^3^H]DAMGO (DAMGO, [Tyr^1^-3,5-^3^H])), [^3^H][Ile^5,6^]DELT II ([Ile^5,6^]Deltorphin II, [Tyr^l^-3,5-^3^H]) and [^3^H]Substance P (Substance P, [Leu-3,4,5-^3^H]), for experiments with MOR, DOR, and NK1R, respectively. The first two were obtained from Isotope Laboratory, Biological Research Centre, Institute of Biochemistry (Szeged, Hungary) [56], whereas the latter was purchased from Perkin Elmer (Perkin Elmer Inc., Krakow, Poland). Membrane fractions of rat brain homogenate were incubated at 25 °C for 60 min in the presence of radioligands (0.5 nM) specific for each receptor and increasing concentrations of the tested compounds. To measure non-specific binding, 10 μM naloxone was used as the competitor for opioid receptors and 10 μM cold Substance P was used for NK1R. The reactions were carried out in assay buffer containing Tris-HCl (pH 7.4) with an addition of bovine serum albumin (BSA) and protease inhibitors (bacitracin, bestatin, captopril). After the incubation, the binding reactions were terminated by rapid filtration with M-24 Cell Harvester (Brandel/USA) through GF/B Whatman glass fiber strips (presoaked with 0.5% PEI in order to minimize non-specific binding). Radioactivity retained on the filters was measured in MicroBeta LS on a Trilux scintillation counter (PerkinElmer, Santa Clara, CA, USA). The experiments were repeated at least two times in duplicate. The IC_50_ value for each compound was determined using GraphPad Prism [57].

### 3.4. Molecular Docking

Prior to molecular docking, the ability of AutoDock 4.2.6 [44] and AutoDock Vina [45] to reproduce the binding pose of DAMGO in the 6DDF structure [43] was checked (results shown in the Supplementary Materials). Since AutoDock 4.2.6. performed better, we chose to focus on this program. 

Molecular docking of the studied compounds to MOR was performed using the local search protocol in AutoDock 4.2.6 [44]. The initial binding poses of the compounds in the MOR binding site were prepared manually by building the *N*-acylhydrazone-, *N*′-acylhydrazide-, or *N*′-acetylhydrazide-based fragments into the peptide structures. The positions of the peptide sequences were based on the experimental positions [43] of corresponding structural elements in DAMGO (**1a**-**d**) or based on the results of molecular modelling [38] for TAPP tetrapeptide amide (**3a** and **3b**) and for other derivatives (**2a–b**, **4a–b, 5a, 6a**). 

The receptor structure was the activated MOR structure (PDB accession code: 6DDF [43]). The G-protein was removed from the receptor structure and the protonation states were set as expected at physiological pH. The ligands and the protein structure were processed in AutoDock Tools 4 [44] following standard routines. The ligands were considered flexible (except for amide bonds of the peptide backbone and the C-terminal part) and the receptor was set as rigid. For modelling of the flexibility of the C-terminal part, several conformers of this part were manually prepared and submitted to the modelling procedure (with the peptide part flexible and the C-terminal organic fragment rigid). The docking box (90 × 104 × 106 points, spaced by 0.375 Å) was set around the position of DAMGO in the 6DDF structure [43] and its size was extended so that it covered the receptor binding pocket and the binding pocket entry. Grids were calculated with AutoGrid. The local search parameters were set as follows: 1000 runs, population of 300 individuals, 500 iterations of Solis and Wets local search, *sw_rho* search space parameter 10.0. The results were clustered and representative poses from several top-scored clusters subject to visual inspection. Molecular graphics were prepared in PyMOL [58].

### 3.5. In Vivo Analgesic Activity after Intrathecal Administration

Analgesic activity of compounds **1b**–**1d** was measured in the tail flick test after intrathecal administration to rats, according to a procedure thoroughly described before [33]. First, adult male Wistar rats weighing 200–250 g were prepared for intrathecal administration by the catherization method described previously by Yaksh and Rudy [59]. Each experimental group consisted of 4–8 rats. Next, spinally mediated analgesia was assessed in the tail flick test utilizing the Plantar Test and Tail Flick Analgesia Meter apparatus (IITC Life Science Inc., Los Angeles, CA, USA). Withdrawal latency was measured in triplicate. The cut-off latencies were set at 7 s to avoid burns. The measurements were performed before the administration of the tested compound (time 0) and 5, 15, 30, 60, and 120 min after injection. The control group received injections of 0.9% NaCl. Data from in vivo studies are presented as means ± SEM and were analyzed using two-way repeated measures of ANOVA followed by Dunnett-corrected multiple comparisons. Significance was defined as * *p* < 0.033, ** *p* < 0.002, *** *p* < 0.001. Data were analyzed using GraphPad Prism [57]. The responses were expressed as a percentage of a maximum possible effect (%MPE), calculated as ((T1 − T0)/(T2 − T0)) × 100, where T0 and T1 are latencies before and after drug injection, respectively, and T2 is the cut-off time. All experimental procedures were approved by The Local Committee for Ethics in Animal Experiments, permission no. 46/2013.

### 3.6. Evaluation of the Compounds’ Influence on Melanoma Cells

The effect that the compounds **1a**–**6a** exert on the melanoma cell number (in cell culture) was tested on a MEW155 human melanoma tumor cell line obtained from the Maria Skłodowska-Curie Memorial Cancer Centre and Institute of Oncology in Warsaw. The cells were plated in 24-well plates (5000 cells per well) and incubated for 24 h in Eagle’s medium containing 10% fetal bovine serum and 1% solution of penicillin and streptomycin. After the cells had reached 80% confluence, the tested compounds (dissolved in 1% DMSO) were added at a concentration of 50 μM. Control cells were treated with 1% DMSO only. After 96 h of incubation, the cell suspension was first lysed in lysis buffer (ReagentA, ChemoMetec A/S) and then stabilized in stabilization buffer (ReagentB, ChemoMetec A/S). The number of dead cells was quantified in a propidium iodide cassette with the use of a NucleoCounter^®^ NC-100™ (ChemoMetec A/S) cell counter. The tests were performed in triplicate. The results were normalized so that the values obtained for the control were 100%.

## 4. Conclusions

In conclusion, the presented research dealt with the question of how the C-terminal extension of short opioid peptide sequences influences the biological properties. The extending moieties we considered were organic fragments consisting of 3,5-bis(trifluoromethyl)phenyl moiety and *N*-acylhydrazone (series **a**) or *N*′-acylhydrazide (series **b**) motifs. Additionally, two compounds (**1c** and **1d**) contained longer elements. Eleven novel analogues (**1b**–**6a**) based on opioid peptides sequences such as enkephalin, TAPP, endomorphin-2, or their truncated variants were synthesized.

The compounds were tested for affinity to MOR, DOR, and NK1R. Regarding the MOR binding, the presented analogues show a diversified range of affinities. Both low nanomolar IC_50_ values (**1c** and **1d**) and values greater than 1000 nM (**4b**) were observed in the set. In three of the four *N*-acylhydrazone–*N*′-acylhydrazide analogue pairs, the hydrazones exhibited better MOR binding (4 to 10-times). The most potent MOR ligands studied here (**1c** and **1d**) contain the longer fragments with *N*′-acetylhydrazide- or -C(=O)-NH-NH-C(=O)-NH-N=CH- motifs. With respect to DOR affinity, most of the novel compounds do not have appreciable binding. Still, the enkephalin-based analogues show DOR affinity that varies depending on the C-terminal fragment. None of the prepared analogues has measurable NK1R binding, except for one compound (**3a**) that showed rather low affinity. The observed experimental MOR affinities were compared to the results of molecular docking. The in silico models provide a rational basis for the design of novel compounds.

Four derivatives based on H-Tyr-D-Ala-Gly-Phe- sequence (**1a**–**1d**) were tested in vivo for antinociceptive activity. All four analogues turned out to be fairly strong analgesics, with the previously published compound **1a** having the lowest ED_50_.

Finally, we have tested the cytotoxic properties of our derivatives (**1a**–**6a**) on the MeW155 human melanoma cell line. At a concentration of 50 µM, the analogues containing the N-acylhydrazone motif significantly affected the cell viability, decreasing the number of cells in the culture. In particular, compounds **3a** and **5a** killed almost all cells in the culture. The impact on cells was not correlated with either opioid or neurokinin-1 receptor affinities. On the other hand, the effect seems rather to be related to the compounds’ lipophilicity.

## Figures and Tables

**Table 1:** Analytical data for the synthesized compounds.

No	Compound	Formula	ESI-MS [M + H] ^+^ *m*/*z*		t_R_ [min]	LogP
			Calcd	Obsvd		
**1a** **[33]**	H-Tyr-D-Ala-Gly-Phe-NH-N=CH-3,5-(CF_3_)_2_Ph	C_32_H_32_F_6_N_6_O_5_	695.24	694.95	12.91	5.55
**1b**	H-Tyr-D-Ala-Gly-Phe-NH-NH-C(=O)-3,5-(CF_3_)_2_Ph	C_32_H_32_F_6_N_6_O_6_	711.24	710.85	11.82	4.5
**1c**	H-Tyr-D-Ala-Gly-Phe-NH-NH-C(=O)-CH_2_-3,5-(CF_3_)_2_Ph	C_33_H_34_F_6_N_6_O_6_	725.25	724.90	12.10	3.54
**1d**	H-Tyr-D-Ala-Gly-Phe-NH-NH-C(=O)-NH-N=CH-3,5-(CF_3_)_2_Ph	C_33_H_34_F_6_N_8_O_6_	753.26	752.85	12.53	4.19
**2a**	H-Tyr-D-Ala-Gly-Trp-NH-N=CH-3,5-(CF_3_)_2_Ph	C_34_H_33_F_6_N_7_O_5_	734.25	733.90	12.88	5.48
**2b**	H-Tyr-D-Ala-Gly-Trp-NH-NH-C(=O)-3,5-(CF_3_)_2_Ph	C_34_H_33_F_6_N_7_O_6_	750.25	749.95	12.03	4.43
**3a**	H-Tyr-D-Ala-Phe-Phe-NH-N=CH-3,5-(CF_3_)_2_Ph	C_39_H_38_F_6_N_6_O_5_	785.29	784.85	14.20	7.74
**3b**	H-Tyr-D-Ala-Phe-Phe-NH-NH-C(=O)-3,5-(CF_3_)_2_Ph	C_39_H_38_F_6_N_6_O_6_	801.28	800.85	13.40	6.69
**4a**	H-Tyr-D-Ala-Trp-NH-N=CH-3,5-(CF_3_)_2_Ph	C_32_H_30_F_6_N_6_O_4_	677.23	677.15	12.94	6.09
**4b**	H-Tyr-D-Ala-Trp-NH-NH-C(=O)-3,5-(CF_3_)_2_Ph	C_32_H_30_F_6_N_6_O_5_	693.23	692.85	12.25	5.04
**5a**	H-Tyr-Pro-Phe-Phe-NH-N=CH-3,5-(CF_3_)_2_Ph	C_41_H_40_F_6_N_6_O_5_	811.30	810.80	14.09	8.03
**6a**	H-Tyr-Pro-Phe-NH-N=CH-3,5-(CF_3_)_2_Ph	C_32_H_31_F_6_N_5_O_4_	664.24	664.16	13.25	6.03

**Table 2:** Affinity of the synthesized compounds for opioid and neurokinin receptors.

	IC_50_ ± SEM ^1^ (nM)		
Compound	MOR	DOR	NK1R
**YaGF-** **(enkephalin-derivative)**			
**1a** [33]	12.73 ± 0.75	74.20 ± 0.40	4.24 µM ± 0.80
**1b**	110.64 ± 12.66	413.45 ± 105.15	>1000
**1c**	5.25 ± 1.14	161.65 ± 8.75	>1000
**1d**	4.73 ± 0.02	44.04 ± 2.75	>1000
**YaGW-** **(enkephalin-derivative)**			
**2a**	112.01 ± 16.89	914.4 ± 43.50	>1000
**2b**	93.465 ± 32.84	467.05 ± 36.95	>1000
**YaFF-** **(TAPP)**			
**3a**	90.4 ± 9.91	>1000	722.8 ± 58.2
**3b**	391.05 ± 57.85	>1000	>1000
**YaW-**			
**4a**	118.11 ± 40.19	621.0 ± 65.20	>1000
**4b**	>1000	>1000	>1000
**YPFF-** **(endomorphin-2)**			
**5a**	28.5 ± 9.41	>1000	>1000
**YPF-** **(truncated endomorphin-2)**			
**6a**	113.1 ± 7.00	>1000	>1000

^1^ IC_50_ values represent means ± SEM of at least two experiments performed in duplicates.

**Table 3:** The effective dose (ED_50_) of compounds **1a**–**1d**.

Cmpd	Compound	ED_50_ ± SEM (nmol/rat) ^1^
**1a**	H-Tyr-D-Ala-Gly-Phe-NH-N=CH-3,5-(CF_3_)_2_Ph	0.18 ± 0.05
**1b**	H-Tyr-D-Ala-Gly-Phe-NH-NH-C(=O)-3,5-(CF_3_)_2_Ph	n./a ^2^
**1c**	H-Tyr-D-Ala-Gly-Phe-NH-NH-C(=O)-CH_2_-3,5-(CF_3_)_2_Ph	0.56 ± 0.04
**1d**	H-Tyr-D-Ala-Gly-Phe-NH-NH-C(=O)-NH-N=CH-3,5-(CF_3_)_2_Ph	0.32 ± 0.04

^1^ ED_50_ (half maximal effective dose) ± standard error of the mean, n = 4–8; ^2^ n/a—not applicable, as there was no dose dependency the value could not be established.

## Supplementary Materials

The following are available online at https://www.mdpi.com/1420-3049/25/15/3429/s1: Figure SM-1. Plot of predicted free energies of binding versus the experimental affinities. Validation of the docking procedure performed with AutoDock 4.2.6 and AutoDock Vina. Table SM-1. Quality of binding pose prediction (Auto Dock 4.2.6). Table SM-2. Quality of binding pose prediction (AutoDock Vina). Results of molecular modelling for selected compounds. Table SM-3. Comparison of docking poses found by AutoDock and AutoDock Vina. NMR assignments for selected compounds.
