# Supplementary material for: Antinociceptive and Cytotoxic Activity of Opioid Peptides with Hydrazone and Hydrazide Moieties at the C-Terminus
Source: Molecules. 2020 Jul 28;25(15):3429. doi: 10.3390/molecules25153429 (PMC7435865; doi:10.3390/molecules25153429)
Supplement: Supplementary file 1 [file molecules-25-03429-s001.pdf]

molecules

Supplementary

Antinociceptive and Cytotoxic Activity of Opioid Peptides with Hydrazone and Hydrazide Moieties at the C-Terminus

Jolanta Dyniewicz [1,*], Piotr F. J. Lipiński [1,*], Piotr Kosson [2], Marta Bochyńska-Czyż [1], Joanna Matalińska [1] and Aleksandra Misicka [1,3,*]

[1] Department of Neuropeptides, Mossakowski Medical Research Centre Polish Academy of Sciences, Pawińskiego 5, 02-106 Warsaw, Poland; marta.bochynska@interia.eu (M.B.-C.); jmatalinska@imdik.pan.pl (J.M.)
[2] Toxicology Research Laboratory, Mossakowski Medical Research Centre Polish Academy of Sciences, Pawińskiego 5, 02-106 Warsaw, Poland; pkosson@imdik.pan.pl
[3] Faculty of Chemistry, University of Warsaw, Pasteura 1, 02-093 Warsaw, Poland; misicka@chem.uw.edu.pl
* Correspondence: jdyniewicz@imdik.pan.pl (J.D.); plipinski@imdik.pan.pl (P.F.J.L.); misicka@chem.uw.edu.pl (A.M.)

Academic Editors: Joanna Bojarska, Wojciech M. Wolf, Milan Remko, Piotr Zielenkiewicz, Michele Saviano, Janusz Zabrocki and Krzysztof Kaczmarek

Received: 01 July 2020; Accepted: 23 July 2020; Published: date

Figure SM-1. Plot of predicted free energies of binding versus the experimental affinities. (Page SM-2)

Validation of the docking procedure performed with AutoDock 4.2.6 and AutoDock Vina. (Page SM-2)

Table SM-1. Quality of binding pose prediction(Auto Dock 4.2.6). (Page SM-3)

Table SM-2. Quality of binding pose prediction (AutoDock Vina). (Page SM-3)

Results of molecular modelling for selected compounds (Page SM-3)

Table SM-3. Comparison of docking poses found by AutoDock and AutoDock Vina. (Page SM-5)

Solvent systems for column chromatography of intermediate compounds. (Page SM-6)

NMR assignments for selected compounds. (Page SM-6)

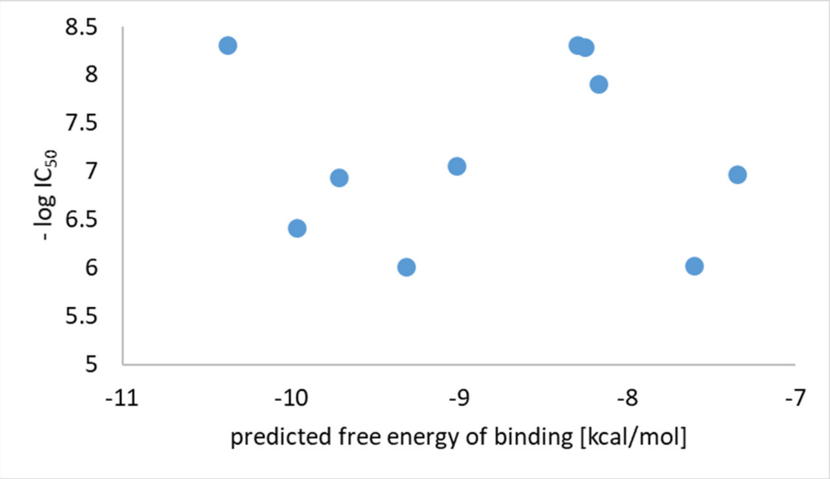

Figure 1. Plot of predicted free energies of binding versus the experimental affinities.

Validation of the docking procedure performed with AutoDock 4.2.6 and AutoDock Vina.

DAMGO was redocked into 6DDF structure (resolution: 3.5 Å) using AutoDock 4.2.6 and AutoDock Vina. The RMSD of the experimental and the predicted positions were calculated. Furthermore, displacement of a few key pharmacophoric elements was calculated, too.

As to AutoDock 4.2.6., the first best scored binding pose was of utterly different orientation than the experimental one (RMSD 5.600 Å), it did not fit our usual criteria for being inconsistent with the mutagenetic data. In particular, the binding mode lacked a canonical interaction between D147 and the protonable amine (displacement: 9.619 Å), therefore it was discarded.

The second best scored binding pose was very close to the experimental one, with RMSD (2.424 Å, for heavy atoms of residues 1-4) lower than resolution of the structure. Positions of protonable nitrogen of Tyr^1, phenolic oxygen of Tyr^1 and the aromatic ring of *N*-$MePhe^4$ were displaced by no more than 1.6 Å from the experimental structure. This is a fairly good result. The fifth residue was placed differently than in the experimental structure, but per our experience with this structure (and the results provided by the authors of the 6DDF structure in the original paper), given that Gly^5-ol does not form strong directional interactions, and that it is quite flexible, it could be expected to retain significant residual flexibility. It is seen in the molecular dynamics simulations (our results unpublished; the results of the original authors given in the paper reporting the 6DDF structure), where this tail is found to be moving over the course of the simulations.

Table 1. Quality of binding pose prediction (AutoDock 4.2.6). All values in Å.

	**Pose 2**	**Pose 1**
**RMSD (heavy atoms, res 1-4)**	2.424	5.60
**Displacements**		
**Tyr[1] nitrogen**	1.560	9.619
**Tyr[1] phenolic O**	1.106	1.943
***N*-MePhe[4] CG**	1.459	5.233

As to AutoDock Vina, none of the 9 best scored docking solutions reported was one that could be considered correct (Table SM-2). Even though, for a few of them, the RMSDs were close to 3 Å, these poses lacked the reproduction of the canonical amine ··· Asp147 interaction. The phenolic oxygen was also placed differently than in the experimental structure. In several instances, Vina correctly placed the *N*-MePhe[4] aromatic ring.

Table 2. Quality of binding pose prediction (AutoDock Vina). All values in Å.

		**Displacement**		
**Binding pose number**	**RMSD**	**Tyr[1] nitrogen**	**Tyr[1] phenolic O**	***N*-MePhe[4] CG**
**1**	3.259	5.421	4.941	0.508
**2**	3.924	5.974	5.378	1.393
**3**	3.111	5.560	4.762	1.334
**4**	8.277	6.534	12.786	6.533
**5**	3.353	5.892	5.803	1.313
**6**	4.540	5.354	9.069	1.124
**7**	9.132	10.813	11.810	9.550
**8**	3.870	3.441	8.987	0.471
**9**	5.933	5.675	6.151	7.575

Results of molecular modelling for selected compounds

1d

The starting position for **1d** local docking was exactly the one that was applied for **1a**-**1c** (based on the experimental position of DAMGO). As with these derivatives, only minor displacement of the N-terminal tetrapeptide was observed as a result of the local search procedure. Regarding the interactions of the C-terminal part, **1d** assumes *cis*-conformation of the first amide bond of the diacylhydrazine motif. This allows it for interacting with Tyr148 via hydrogen bond between the phenolic oxygen and hydrogen of this amide. The elements of the hydrazone substructure are predicted not to be involved in any contacts with the receptor. One of the CF_3 groups forms hydrophobic interactions with Thr218 and Leu219.

2. a and 2b

The starting position for compounds **2a** and **2b** was prepared based on the DAMGO experimental position. The *N*Me-Phe[4] was replaced for Trp[4] and the appropriate C-terminal elements were added. The bulky Trp side chain causes the tetrapeptide to modify its positioning (Figure SM-2). This does not influence the interactions of the N-terminal part. The canonical ionic contact between Tyr1 amine and Asp147 is present in the predicted binding modes. One can observe furthermore that the phenol group of Tyr1 is located similarly as the phenol of DAMGO in the 6DDF structure. The Trp[4] indole ring is placed in the hydrophobic subpocket made of side chains of several residues belonging to transmembrane helix 3 (TM3) and extracellular loops 1 and 2 (ECL1 and ECL2). AutoDock predicts that the indole's N-H can be involved in hydrogen bond to Cys217 carbonyl group. As to the C-terminal part, in the hydrazone derivative (**2a**), contrary to what was predicted for **1a,** there is no hydrogen bond between the hydrazone's amide and Tyr148. This is probably due to the fact that the bulky Trp[4] requires displacing slightly the whole structure. The lack of this interaction corresponds to lower binding affinity of **2a** compared to **1a**. In the *N′*-acylhydrazide

derivative (2b), this hydrogen bond is present and the 3,5-*bis*-trifluoromethylphenyl group approaches TM7, forming some hydrophobic contacts with Trp318.

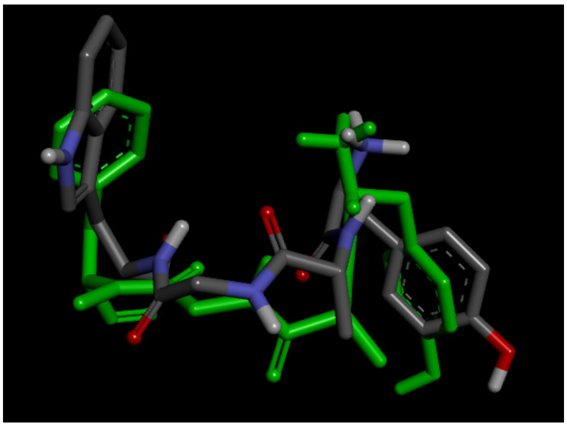

Figure 2. Binding mode of compound **2b** (residues 1-4) compared to the position of DAMGO in 6DDF (green sticks).

5a

The starting position for compound **5a** was obtained by docking endomorphin-2 (Tyr-Pro-Phe-Phe-NH_2) to the mu-opioid receptor (6DDF). We have docked the EM-2 instances with both *cis*- and *trans*-conformation of the Tyr-Pro peptide bond. AutoDock 4.2.6 predicted better interaction energy (and a reasonable binding mode) for the instance with the *cis*-conformation. This binding mode was chosen for building the **5a** structure that was further subject to local docking.

The complex μOR ··· 5a is predicted to be stabilized by the following interactions:

a) the canonical ionic interaction of Tyr^1 amine with Asp147,
b) hydrophobic interactions of Pro^2 ring with Trp318 and Ile322,
c) interactions of the Phe^3 ring with the Val143, Trp133 (positioning similar to the positioning of Phe^4 in enkephalins),
d) hydrogen bonds of Asp216 with the hydrazone motif (Figure SM-3),
e) hydrophobic contacts of Thr218, Lys209 with the 3,5-*bis*-trifluoromethylphenyl group.

Figure SM-3. A general glance at the binding mode of 5a. The ligand is shown as sticks. The receptor is represented by helices. Only several side chains are shown. Asp216 side chain is marked as sticks and the hydrogen bond between the side chain and the hydrazone motif is shown.

6a

The starting position for compound **6a** was obtained by docking tripeptide amide (Tyr-Pro-Phe-NH_2) to the mu-opioid receptor (6DDF). Again, both *cis*- and *trans*-conformations of the Tyr-Pro peptide bond were taken into account, and again, the *cis*-conformation was predicted to have a higher interaction energy. The obtained binding mode served for building the **6a** structure that was input for local docking procedure. The computed interactions of the residues are identical as in the case of compound **5a**. No contacts are predicted for the hydrazone motif. The 3,5-bis-trifluoromethylphenyl group approaches TM6 and TM5 and forms interactions with the side chain of Glu229 and Lys303.

Table 3. Comparison of docking poses found by AutoDock and AutoDock Vina. All values in Å.

Compound	RMSD (heavy atoms) (AutoDock vs AutoDock Vina)
**1a**	2.416
**1b**	1.320
**1c**	1.200

**1d**	2.144
**2a**	4.226
**2b**	3.577
**3a**	1.785
**3b**	5.125
**4a**	0.885
**4b**	1.679
**5a**	3.495
**6a**	5.055

Solvent systems for column chromatography of intermediate compounds.

Compound Boc-Tyr-D-Ala-Gly-Trp-NH-N=CH-3,5-$(CF_3)_2$Ph
Solvents system ethyl acetate/methanol in ratio 9:1. Yield after purification 21%.
Compound Boc-Tyr-D-Ala-Phe-Phe-NH-N=CH-3,5-$(CF_3)_2$Ph
Solvents system ethyl acetate/methanol in ratio 95:5. Yield after purification 28%.
Compound Boc-H-Tyr-D-Ala-Trp-NH-N=CH-3,5-$(CF_3)_2$Ph
Solvents system ethyl acetate/methanol in ratio 9:1. Yield after purification 59%.
Compound Boc-Tyr-Pro-Phe-Phe-NH-N=CH-3,5-$(CF_3)_2$Ph
Solvents system ethyl acetate/hexane in ratio 9:1. Yield after purification 32%.
Compound Boc-Tyr-Pro-Phe-NH-N=CH-3,5-$(CF_3)_2$Ph
Solvents system ethyl acetate/methanol in ratio 8:2. Yield after purification 37%.
Compound Boc-Tyr-D-Ala-Gly-Phe-NH-NH-C(=O)-3,5-$(CF_3)_2$Ph
Solvents system ethyl acetate/methanol in ratio 95:5. Yield after purification 16%.
Compound Boc-Tyr-D-Ala-Gly-Phe-NH-NH-C(=O)-CH_2-3,5-$(CF_3)_2$Ph
Solvents system ethyl acetate/methanol in ratio 99:1. Yield after purification 22%.
Compound Boc-Tyr-D-Ala-Gly-Phe-NH-NH-C(=O)-NH-N=CH-3,5-$(CF_3)_2$Ph
Solvents system ethyl acetate/methanol in ratio 9:1. Yield after purification 20%.
Compound Boc-Tyr-D-Ala-Gly-Trp-NH-NH-C(=O)-3,5-$(CF_3)_2$Ph
Solvents system ethyl acetate/methanol in ratio 95:5. Yield after purification 19%.
Compound Boc-Tyr-D-Ala-Phe-Phe-NH-NH-C(=O)-3,5-$(CF_3)_2$Ph
Solvents system ethyl acetate/methanol in ratio 95:5. Yield after purification 42%.
Compound Boc-Tyr-D-Ala-Trp-NH-NH-C(=O)-3,5-$(CF_3)_2$Ph
Solvents system ethyl acetate/methanol in ratio 99:1. Yield after purification 20%.

NMR assignments for selected compounds:

Compound **2b** (H-Tyr-D-Ala-Gly-Trp-NH-NH-C(=O)-3,5-$(CF_3)_2$Ph)

^{1}H NMR(600 MHz, DMSO) δ: 10.80 (1Hε1, Trp) 10.55 (d, J=2.32 Hz, 2H, NH-NH), 9.35 (br. s, 1H, OH, Tyr), 8.52 (1H, NH, Ala), 8.51 (1H, Ar), 8.41 (s, 1H, Ar), 8.28 (d, J=8.10 Hz, 2H, NH-Tyr), 8.21 (d, J=8.10 Hz, 1H, NH, Trp), 8.18 (1H, NH, Gly), 7.68 (d, J=7.8 Hz, 1Hε3, Trp), 7.35 (d, J=8.1 Hz, 1Hζ2, Trp), 7.24 (1Hδ1, Trp), 7.08 (t, J=7.05, 1Hη, Trp), 7.02 (1Hζ3-Trp), 7.01 (d, J=6.27 Hz, 2Hδ-Tyr), 6.70 (d, J=6.53 Hz, 2Hε-Tyr), 4.70 (J=4.7 Hz, 1Hα-Trp), 4.33 (t, J=7.32 Hz, 1Hα-Ala), 3.9 (J=6.79 Hz, 1Hα-Tyr), 3.76 (d, J=5.75 Hz, 1Hα-Gly), 3.63 (d, J=5.75 Hz, 1Hα-Gly), 3.23 (d, J=3.92 Hz, 1Hβ–Trp), 3.21 (d, J=3.92 Hz, 1Hβ-Trp), 2.92 (1Hβ–Tyr), 2.88 (1Hβ–Tyr), 1.06 (d, J=6.79 Hz, 3Hβ-Ala),

^{13}C NMR(150 MHz, DMSO) δ: 175.0 (C=O, Ar), 172.0 (C=O, Ala), 168.7 (C=O, Gly), 167.9 (C=O, Tyr), 156.7 (Cζ–Tyr), 136.15 (Cε2–Trp), 130.6 (2Cδ–Tyr), 128.6 (CAr), 127.5(Cδ2–Trp), 126.1 (CAr), 125.1 (C–Tyr), 124.5 (Cδ1–Trp), 124.4 (CAr), 122.2 (CAr), 121.2 (Cη2–Trp), 118.7 (Cε3,ζ2–Trp), 115.5 (Cε–Tyr), 111.7 (Cζ2–Trp), 109.8 (C–Trp), 54.6 (Cα, Tyr), 52.3 (Cα, Trp), 48.3 (Cα, Ala), 41.1 (Cα, Gly), 36.7(Cβ, Tyr), 28.5 (Cβ, Phe), 18.6 (Cβ, Ala).

Compound **3a** (H-Tyr-D-Ala-Phe-Phe-NH-N=CH-3,5-$(CF_3)_2$Ph)

^{1}H NMR(600 MHz, DMSO) δ: 9.35 (1H, OH-Tyr), 8.03-8.88 (3H, Ar, 1H, N=CH, 1H, NH, Ala, 2H, NH, Tyr, 2H, NH, Phe2,3, 1H, NH-N), 7.15-7.32 (m, 10Hδ,ε,ζ, Phe2,3, 1H, Ar), 6.97 (2Hδ1,δ2 ,Tyr), 6.67 (m, 2Hε1,ε2,Tyr), 4.57-4.69 (m, 2Hα,Phe2,3), 4.35 (m, 1Hα, Ala), 3.94 (m, 1Hα, Tyr), 3.05-3.12 (m, 2Hβ, Phe2,3, 1Hβ, Tyr), 2.79-2.88 (m, 2Hβ, Phe2,3, 1Hβ, Tyr), 1.09 (d, J= 7.02 Hz, 3Hβ, Ala).

Compound **3b** (H-Tyr-D-Ala-Phe-Phe-NH-NH-C(=O)-3,5-$(CF_3)_2$Ph)

^{1}H NMR(600 MHz, DMSO) δ: 10.48 (2H, NH-NH), 9.34 (br. s, 1H, OH-Tyr), 8.79 (2H, NH, Phe2,3), 8.55 (1H, Ar), 8.41 (s, 1H, Ar), 8.34 (d, J=8.24, 1H, NH, Tyr), 8.29 (d, J=9.16, 1H, NH, Ala), 7.40 (1Hζ, Phe), 7.31 (m, J=7.63 Hz, 2Hε, Phe), 7.29 (m,J=7.63 Hz, 2Hd, Phe), 7.24 (1H, Ar), 7.18 (d, J=7.32 Hz, 1Hζ, Phe), 7.15 (m, J=7.32 Hz, 2Hε, Phe), 7.10 (m, J=7.32 Hz, 2Hε, Phe), 6.98 (d, J=7.93 Hz, 2Hδ, Tyr), 6.67 (d, J=8.24 Hζ, 2Hε, Tyr), 4.79 (m, 1Hα, Phe), 4.62 (m, 1Hα, Phe), 4.34 (k, J=7.02 Hz, 1Hα, Ala), 3.94 (1Hα, Tyr), 3.14 (2Hβ, Phe2,3), 2.73-2.86 (2Hβ, Phe2,3, 2Hβ, Tyr), 1.05 (d, J=7.02 Hz, 3Hβ, Ala).

Compound **4b** (H-Tyr-D-Ala-Trp-NH-NH-C(=O)-3,5-(CF3)2Ph)

^{1}H NMR(600 MHz, DMSO) δ: 10.85 (1Hε1–Trp), 10.53 (2H, NH-NH), 9.35 (br. s, 1H, OH, Tyr), 8.52 (1H, NH), 8.50 (1H, Ar), 8.42 (s, 1HAr), 8.28 (1H, NH), 8.15 (1H, NH), 7.72 (1H, Trp), 7.34 (1H, Trp), 7.25 (1H, Trp), 7.07 (1H, Trp), 7.05 (1H, Trp), 7.00 (2Hδ-Tyr), 6.69 (m, 2Hε, Tyr), 4.78 (m, 1Hα, Trp), 4.38 (t, J=7.17 Hz, 1Hα, Ala), 3.97 (m, 1Hα, Tyr), 3.22 (1Hβ, Trp), 3.20 (d, J=3.2 Hz, 1Hβ, Trp), 2.97 (1Hβ, Tyr), 2.85 (1Hβ, Tyr), 1.06 (d, J=6.7 Hz, 3Hβ, Ala).

Compound **5a** (H-Tyr-Pro-Phe-Phe-NH-N=CH-3,5-(CF3)2Ph)

^{1}H NMR(600 MHz, DMSO): δ: 9.36 (1H, OH,Tyr), 8.56 (1H, -NH-N=),8.45 (s, 1HAr), 8.35-8.36 (2H, NH-Phe3, Phe4), 8.33 (NH-Tyr), 8.17 (s, 1H, N=CH), 8.15 (s, 1HAr), 8.02 (s,1HAr), 7.15-7.29 (10Hδ,ε,ζ–Phe3, Phe4), 6.89-6.91 (2Hδ–Tyr), 6.68-6.71 (2Hε–Tyr), 4.59 (1Hα–Tyr),4.39 (1Hα–Pro), 3.59 (2Hδ–Pro),3.24 (2Hα–Phe1,Phe2), 3.07 (1Hβ–Tyr), 2.91-2.95 (2Hβ–Phe1, Phe2), 2.77-2.85 (2Hβ–Phe3,Phe4), 2.79 (1Hβ– Tyr),1.92-2.01 (3Hβ,γ–Pro), 1.75 (1Hγ– Pro).
